# Supplementary material for: Impact of Q-fever on physical and psychosocial functioning until 8 years after Coxiella burnetii infection: An integrative data analysis
Source: PLoS One. 2022 Feb 2;17(2):e0263239. doi: 10.1371/journal.pone.0263239 (PMC8809529; doi:10.1371/journal.pone.0263239)
Supplement: S1 Appendix — (DOCX) [file pone.0263239.s001.docx]

***Supplementary material***

| Table 4 Results from the sensitivity analysis in three multilevel linear regression models per Q-fever group and outcome corrected for gender. Basic model with all data, sensitivity model 1 (excluding time points baseline and 8 years) and sensitivity model 2 (excluding time points baseline, 3, 5 and 8 years) | | | | | | | | |  |
| --- | --- | --- | --- | --- | --- | --- | --- | --- | --- |
|  |  | **Intercept: score at baseline** | | |  | **Slope: change in score (per year)** | | | |
|  |  | QFS | Chronic  Q-fever | past acute  Q-fever |  | QFS | Chronic  Q-fever | past acute  Q-fever | |
|  |  | β (95% CI) | β (95% CI) | β (95% CI) |  | β (95% CI) | β (95% CI) | β (95% CI) | |
| Basic model |  |  |  |  |  |  |  |  | |
| Fatigue^1^ |  | 45.8 ( 43.3; 48.3 ) ^a^ | 35.6 ( 30.5; 40.8 ) ^b^ | 37.1 ( 35.8; 38.3 ) ^b^ |  | -0.18 ( -0.75; 0.39 ) ^a^ | 0.52 ( -0.5; 1.53 ) ^a^ | -0.91 ( -1.23; -0.59 ) ^b^* | |
| Quality of Life^2^ |  | 72.2 ( 68.5; 75.9 ) ^a^ | 87.4 ( 81.5; 93.3 ) ^b^ | 84.9 ( 83.5; 86.3 ) ^b^ |  | 0.28 (-0.34; 0.90 ) ^a^ | -1.4 (-2.37; -0,43 ) ^b^* | 0.12 ( -0.19; 0.43 ) ^a^ | |
| Physical Impairment^1^ |  | 13.9 ( 12.0; 15.8 ) ^a^ | 12.6 ( 8.2; 16.9 ) ^a,b^ | 8.4 ( 7.3; 9.4 ) ^b^ |  | 0.51 ( 0.14; 0.87 ) ^a^* | 1.28 ( 0.54; 2.02 ) ^a^* | -0.06 ( -0.32; 0.19 ) ^b^ | |
| Social Participation^2^ |  | 50.5 ( 43.1; 57.8 ) ^a^ | 44.6 ( 36.1; 53.0 ) ^a^ | 68.5 ( 64.5; 72.5 ) ^b^ |  | 0.12 ( -1.20; 1.40 ) ^a^ | 1.1 ( -0.54; 2.80 ) ^a^ | 5.20 ( 3.30; 7.20 ) ^b^* | |
|  |  |  |  |  |  |  |  |  | |
| Sensitivity model 1 |  |  |  |  |  |  |  |  | |
| Fatigue^1^ |  | 45.5 ( 42.8; 48.2 ) ^a^ | 35.7 ( 30.1; 41.2 ) ^b^ | 36.4 ( 35.2; 37.7 ) ^b^ |  | -0.09 ( -0.77; 0.59 ) ^a^ | 0.48 ( -0.71; 1.68 ) ^a^ | -0.74 ( -1.07; -0.41 ) ^a^* | |
| Quality of Life^2^ |  | 71.1 ( 66.9; 75.2 ) ^a^ | 87.5 ( 81.1; 93.8 ) ^b^ | 84.7 ( 83.3; 86.1 ) ^b^ |  | 0.57 ( -0.18; 1.32 ) ^a^ | -1.38 ( -2.48; -0.29 ) ^b^* | 0.16 ( -0.15; 0.47 ) ^a^ | |
| Physical Impairment^1^ |  | 13.8 ( 11.7; 15.9 ) ^a^ | 14.1 ( 9.3; 18.9 ) ^a^ | 8.1 ( 7.0; 9.2 ) ^b^ |  | 0.54 ( 0.12; 0.97 ) ^a^* | 1.14 ( 0.26; 2.02 ) ^a^* | 0.01 ( -0.26; 0.27 ) ^b^ | |
| Social Participation^2^ |  | 48.9 ( 41.0; 56.8 ) ^a^ | 44.2 ( 35.4; 52.9 ) ^a^ | 71.5 ( 66.9; 76.1 ) ^b^ |  | 0.67 ( -0.90; 2.20 ) ^a^ | 1.30 ( -0.57; 3.10 ) ^a^ | 3.20 ( 0.80; 5.60 ) ^a^* | |
|  |  |  |  |  |  |  |  |  | |
| Sensitivity model 2 |  |  |  |  |  |  |  |  | |
| Fatigue^1^ |  | 45.0 ( 42.2; 47.9 ) ^a^ | 35.6 ( 30.0; 41.1 ) ^b^ | 36.6 ( 35.3; 37.9 ) ^b^ |  | -0.02 ( -0.75; 0.72 ) ^a,b^ | 0.53 ( -0.72; 1.78 ) ^a^ | -0.80 ( -1.13; -0.46 ) ^b^* | |
| Quality of Life^2^ |  | 71.1 ( 66.9; 75.4 ) ^a^ | 87.2 ( 80.6; 93.7 ) ^b^ | 84.8 ( 83.4; 86.1 ) ^b^ |  | 0.64 ( -0.12; 1.40) ^a^ | -1.38 ( -2.52; -0.25 ) ^b^* | 0.19 ( -0.12; 0.51) ^a^ | |
| Physical Impairment^1^ |  | 13.3 ( 11.0; 15.5 ) ^a^ | 12.5 ( 7.5; 17.6 ) ^a,b^ | 8.1 ( 7.0; 9.2 ) ^b^ |  | 0.53 ( 0.07; 0.99 ) ^a,b^* | 1.57 ( 0.63; 2.51 ) ^a^* | 0.01 ( -0.25; 0.27 ) ^b^ | |
| Social Participation^2^ |  | 50.7 ( 42.5; 58.9 ) ^a^ | 42.2 ( 33.1; 51.3 ) ^a^ | 71.2 ( 66.8; 75.5 ) ^b^ |  | 0.50 ( -1.10; 2.10 ) ^a^ | 0.97 ( -0.95; 2.90 ) ^a^ | 3.10 ( 1.05; 5.20 ) ^a^* | |
| a,b,c The same superscript letter in each row denotes which intercept (score at baseline) does not differ significantly between Q-fever groups, based on overlapping 95% CI, and which slope (score per time point) does not differ significantly between Q-fever groups by testing the significance of fixed effects at the 0.05 level. Consequently, different letters represent significant differences.  * Change in slope (score per year) is significant at the 0.05 level (every intercept is significant at the 0.05 level)  1 Higher scores mean higher levels of fatigue; more physical impairment. A positive slope indicates deterioration, i.e. an increase in levels of fatigue, or an increase in physical impairment.  2 Higher scores mean higher levels (better) quality of life or social participation. A positive slope indicates an improvement, i.e. an increase in quality of life, or an increase in social participation.  QFS = Q-fever fatigue syndrome; CI = confidence interval. | | | | | | | | |  |
